# Diabetes mellitus as a multisystem disease: understanding subtypes, complications, and the link with steatotic liver diseases in humans

**DOI:** 10.1007/s42000-025-00701-y

**Published:** 2025-08-01

**Authors:** Anna Giannakogeorgou, Michael Roden, Kalliopi Pafili

**Affiliations:** 1 Institute of Clinical Diabetology, German Diabetes Center, Leibniz Institute for Diabetes Research at Heinrich Heine University, Düsseldorf, Germany; 2 German Center for Diabetes Research (DZD E.V.), Partner Düsseldorf, Munich-Neuherberg, Germany; 3 Department of Endocrinology and Diabetology, Faculty of Medicine and University Hospital, Heinrich Heine University, Moorenstraße 5 40225, Düsseldorf, Germany

**Keywords:** Diabetes mellitus, Diabetes subtypes, Clustering, Insulin resistance, Fatty liver, MASLD, Cardiovascular diseases, Neuropathy

## Abstract

**Background & scope of review:**

Diabetes mellitus encompasses a spectrum of metabolic disorders characterized by hyperglycemia. The currently most replicated phenotypic clustering approach, introduced by Ahlqvist et al. and validated by Zaharia et al., identified subtypes based on clinical presentation and underlying pathophysiology. This classification aims at predicting complication risk and enabling targeted therapies. Our review explores shared and distinct mechanisms driving complications, focusing on cardiovascular disease and metabolic dysfunction-associated steatotic liver disease (MASLD), both strongly linked to insulin resistance. We also summarize treatment strategies targeting both conditions and outline mechanisms specific to the development of diabetic foot syndrome, exemplifying the continuum from localized to systemic complications.

**Methods:**

We conducted a narrative review of human and translational studies, focusing on mechanisms and treatments across the above phenotype-based diabetes subtypes, given their reproducibility across populations.

**Main results:**

Diabetes is a multisystem disorder involving a cascade of metabolic disturbances. These include mitochondrial adaptations in key metabolically active tissues contributing to systemic and tissue-specific insulin resistance. Inflammation, inadequate immune responses, oxidative stress, and genetic and environmental factors shape the development of comorbidities whose prevalence varies across subtypes. The interplay between MASLD and diabetes forms a vicious cycle of metabolic abnormalities. Novel treatments show promise in both liver and glycemic endpoints.

**Conclusion:**

Phenotype-based diabetes subtypes exhibit distinct underlying pathophysiological mechanisms which shape the development of complications, with insulin resistance serving as the central link. Targeting these pathways can pave the way for personalized diabetes therapies.

**Graphical Abstract:**

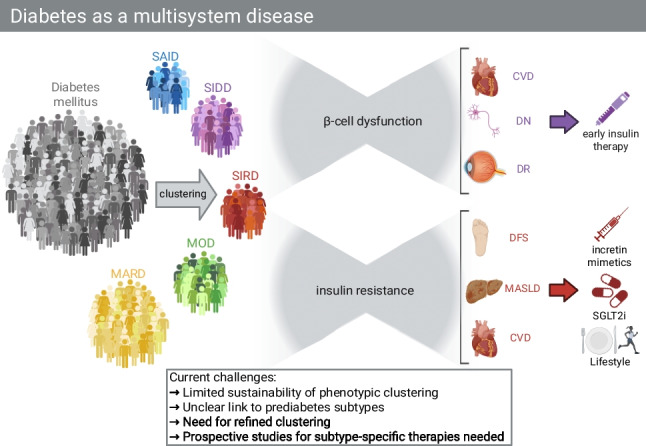

## Introduction

Diabetes mellitus, which is defined by increased blood glucose, comprises a wide range of metabolic disorders affecting carbohydrate metabolism characterized by inefficient use and excessive production of glucose [1]. With over 10.5% of the global adult population being affected, diabetes does not only represent a major health challenge but also a global health-care and economic burden [2].

Traditionally, diabetes has been divided into type-1 (T1D) and type-2 (T2D) diabetes [1], the division primarily based on the presence (T1D) or absence (T2D) of autoantibodies against pancreatic islet β-cells and age at time of diagnosis [3].

While this simplistic classification into T1D and T2D has shaped clinical practice and decision-making for decades, the need for a more nuanced classification has become increasingly apparent, as the multitude of pathophysiological alterations—and networks thereof [4]—and the involvement of various factors such as genetics, environment, age, obesity, and metabolic dysfunction, which influence the prognosis and management of T2D, are increasingly recognized [5]. More recently, a paradigm shift has occurred steering away from defining diabetes through hyperglycemia as a symptom and more toward a precision medicine-orientated approach focusing on diabetes subtypes (also termed endotypes) based on clinical phenotype and the predominant underlying pathophysiological features. The currently most replicated approach was introduced by Ahlqvist et al. [3] and later validated by Zaharia et al.[6]. These subclassifications aim to address the heterogeneity of diabetes, enable early identification of populations at increased risk for complications, and guide timely treatment initiation [3].

Notably, subtypes are not only distinct in their pathophysiology but also differ in prevalence, mechanisms, and risk for development of diabetes-related complications [5, 6], including but not limited to the liver and cardiovascular system driven primarily by chronic hyperglycemia [7]. Distinct mechanisms contribute to the development of diabetic foot syndrome (DFS) across all diabetes subtypes and shape its prevention strategies. However, insulin secretion and sensitivity as well as the presence of complications at time of diagnosis vary significantly even long before the development of hyperglycemia, hinting at different pathophysiological mechanisms driving the development and progression of these systemic disorders [8]. Furthermore, these organ systems and their inter-organ communication (crosstalk) not only appear to be affected by diabetes but may also contribute to its development [8].

In this review, we will first describe the novel concept of diabetes subtypes and their relation to complications, exploring the distinct pathophysiological mechanisms contributing to the development of diabetes-related complications across subtypes, with an emphasis on cardiometabolic and end-organ complications, and, most importantly, metabolic-dysfunction steatotic liver disease (MASLD). We will also discuss available therapeutic strategies for MASLD, including lifestyle-based and pharmacological approaches and how these may also aid in the management of diabetes and its complications for each subtype.

## Introducing phenotype-based diabetes subtypes and their risks for complications

**Fig. 1:**
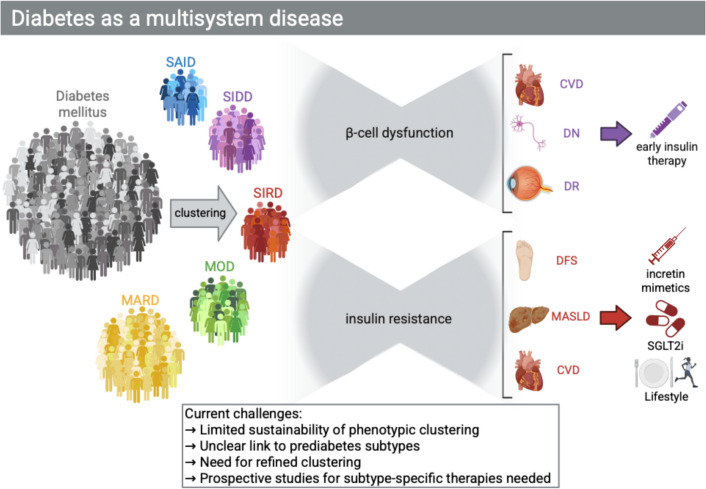
Diabetes as a multisystem disease: toward subtype-specific therapies. Graphical overview highlighting the challenges of current phenotype-based clustering approaches as proposed by Ahlqvist et al., key pathophysiological features linking subtypes to specific complications and their overlaps, and the need for prospective studies to guide the development of tailored, subtype-specific therapeutic strategies. Severe insulin-deficient diabetes (SIDD) and severe autoimmune diabetes (SAID) are primarily characterized by β-cell dysfunction and are strongly associated with diabetic retinopathy (DR), diabetic nephropathy (DN), and cardiovascular disease (CVD). These subtypes often necessitate early insulin therapy. Conversely, severe insulin-resistant diabetes (SIRD) and mild obesity-related diabetes (MOD) are driven mainly by insulin resistance, which links them to their complications such as cardiovascular disease (CVD), metabolic dysfunction-associated steatotic liver disease (MASLD), and diabetic foot syndrome (DFS). Current therapeutic strategies target insulin resistance and its related complications through incretin mimetics, SGLT2 inhibitors (SGLT2i), and lifestyle interventions including diet and exercise. Prospective studies are needed to refine subtype definitions and support the development of precision medicine approaches tailored to each subtype

Various clustering methods—such as k-means, hierarchical, and latent-class trajectory analysis—have been used to subgroup individuals with diabetes based on clinical, genetic, or omics data [1, 9–12]. While some methods rely on high-dimensional electronic health records or genetic risk scores, others, like the widely replicated Ahlqvist model, use clinically accessible variables. Although omics-based and genetic classifications offer mechanistic insights, their clinical utility remains limited, whereas phenotype-based subtypes offer reproducibility and direct applicability in clinical care. In this review, we will focus on the phenotype-based clustering introduced by Ahlqvist et al. [3], later validated by Zaharia et al., using gold-standard methods for metabolic phenotyping [6] and reproduced in many populations globally [5] due to its clinical applicability and utilization of clinically available clustering parameters (Fig. 1).

These approaches have subclassified individuals based on commonly used and easily determined clinical variables for people with diabetes, namely, age at manifestation of diabetes, body mass index (BMI), glycated hemoglobin (HbA1c), homeostasis model assessment of β-cell function (HOMA2-B), and homeostasis model assessment of insulin resistance (HOMA2-IR) and glutamic acid decarboxylase antibodies (GADA) [3, 6]. The severe autoimmune diabetes (SAID) cluster is distinguished by the presence of GADA and most closely resembles T1D [3]. A relatively early onset, impaired insulin secretion, low BMI, and high HbA1c are characteristic of this cluster [3]. The severe insulin-deficient diabetes (SIDD) subtype presents with similar characteristics as the SAID subtype, however, without the presence of GADA autoantibodies [3]. The severe insulin-resistant diabetes (SIRD), most closely mirroring the traditional less well controlled T2D, is characterized by high BMI but low HbA1c as well as impaired insulin secretion and insulin resistance, as reflected by elevated HOMA2-B and HOMA2-IR [3]. Mild obesity-related diabetes (MOD) was related to high BMI but not significant insulin resistance [5]. Lastly, mild age-related diabetes (MARD) was distinguished by a late-onset of diabetes[3].

Based on this recent diabetes classification, SAID, SIDD, and SIRD represent the most aggressive subtypes, requiring intensive treatment interventions for distinct reasons. SAID, most closely resembling T1D, characterized by rapidly progressing β-cell failure, and SIDD, sharing features with SAID but of non-autoimmune origin, require immediate insulin therapy and aggressive glucose monitoring as they are characterized by poor glycemic control [5, 9]. SIRD, distinguished by insulin resistance and, thus, a high comorbidity burden, also demands aggressive treatment involving a combination of strategies targeting glycemic control and organ protection due to its rapid progression and elevated risk of complications [9]. Taken together, these various means of early stratification have major implications for choice of treatment, with SAID and SIDD warranting the most urgent intervention due to rapid glycemic deterioration, while SIRD necessitates targeted management of metabolic dysfunction and insulin resistance.

However, it is important to note that depending on initial cluster allocation, 10–25% of individuals switch cluster allocation over time, a phenomenon linked to age, obesity, and medications affecting body weight and glycemia [6].

### Diabetes subtypes and risk stratification

With regard to glycemia, the SAID and SIDD clusters had, as aforementioned, the highest HbA1c at the time of diagnosis, which resulted in earlier initiation of insulin treatment compared to other clusters [5]. Additionally, the SIDD group had the highest prevalence of retinopathy [5], polyneuropathy, and cardiac autonomic neuropathy at time of diagnosis, which showed no improvement at 5-year follow-up despite improved glycemic control [6]. Indeed, retinopathy prevalence and severity differ across subtypes, with SIDD and SAID showing the highest risk due to early β-cell failure and poor glycemic control. A recent review provides an in-depth overview of retinopathy in diabetes subtypes, confirming these trends and their clinical implications [13]. Moreover, this subcluster exhibited a higher risk for diabetic foot compared to other clusters [14] (Table 1). The SIRD subgroup exhibited the highest risk for diabetic kidney disease (DKD) [3, 6], with evidence showing that eGFR was the lowest of all groups both at time of diagnosis and at 5-year follow-up [6]. The increased DKD risk in SIRD was unrelated to age, sex, and baseline eGFR [3] (Table 1).

**Table 1:** Diabetes subtypes and associated complications in humans: a mechanistically referenced synthesis of cluster-based, peer-reviewed studies

Complication	Cluster most affected	Studies linking clusters to complications	Populations studied	Risk factors	Key mechanistic insights	Studies on mechanisms
CVD	SIDD	Kahkoska et al. (2020) [15]	Asian European North American	↓ BMI	B-cell dysfunction; mechanisms not directly driven by IR or hyperinsulinemia	Kahkoska et al. (2020) [15]
DKD	SIRD	Ahlqvist et al. (2018) [3] Zaharia et al. (2019) [6] Zaharia et al. (2024) [16] Christensen et al. (2022) [17]	European European European European	↑ IR ↑ BMI ↓ FFM Smoking ↓ Physical activity Visceral adiposity ↑ IHL MASLD PNPLA3 rs738409(G) Dysbiosis	IR in CKD is related to chronic inflammation, OS and endothelial dysfunction IR deteriorates renal hemodynamics through SNS activation, sodium retention and downregulation of the natriuretic peptide system IR accelerates progression of CKD/DKD to ESRD, as well as development and progression of CVD Cardiorenal mechanisms involving right ventricular hypertrophy, endothelial dysfunction and atherosclerosis Defective renal clearance in CKD/DKD further impairs hyperinsulinemia in the presence of IR Long-term hemodialysis significantly improves IR IR in CKD/DKD is more closely related to impaired glucose uptake by skeletal muscle rather than the liver	Spoto et al. (2016) [18]
					↑Markers of inflammation and endothelial cell adhesion (IL-6) in PNPLA3 G-allele carriers ↑Systemic inflammation (hsCRP)	Zaharia et al. (2024) [16]
					Treatment with the endothelin receptor antagonist atrasentan shows the greatest improvements in IR in the SIRD cluster, accompanied by a reduction of kidney outcomes, highlighting the pivotal role of IR in DKD development	Smeijer et al. (2025) [19]
	SIDD	Kahkoska et al. (2020) [15]	Asian European North American		↑ HbA1c and lipid levels promote the development of microvascular complications such as DKD	Kahkoska et al. (2020) [15]
					RAAS dysregulation (↑ angiotensin II) and impaired tubulo-glomerular feedback result in ↑ intraglomerular pressure leading to glomerular hyperfiltration and DKD progression ↑AGE accumulation due to chronic hyperglycemia exacerbate OS and fibrosis and activate an inflammatory responses through NF-κB and ROS	Mazzieri et al. (2024) [20]
MASLD	SIRD	Ahlqvist et al. (2018) [3] Christensen et al. (2022) [17] Zaharia et al. (2019) [6] Zaharia et al. (2024) [16]	European	↑BMI ↑IR PNPLA3 status	↓Whole-body insulin sensitivity (M-value, HOMA2-IR)	Ahlqvist et al. (2018) [3]
			European European		↑NEFA ↑Adipo-IR	Zaharia et al. (2019) [6] Zaharia et al. (2020) [21]0000–00-00 00:00:00
			European	↑IR PNPLA3 rs738409(G)	↑Inflammation (hsCRP, IL-6)	Zaharia et al. (2024) [16]
DSPN	SIDD	Zaharia et al. (2019) [6] Ahlqvist et al. (2018) [3]	European European	↑ Disease duration ↑ Age ↑ HbA1c	Lower insulin secretion was related to higher DSPN scores	Schön et al. (2024) [4]
CAN	SIDD	Zaharia et al. (2019) [6] Schön et al. (2024) [4]	European European	↑ HbA1c ↑ Disease duration ↑ Age Smoking	Low insulin secretion was associated with ↑ CAN risk	Schön et al. (2024) [4]
DR	SIDD	Ahlqvist et al. (2018) [3] Anjana et al. (2020) [22] Scott et al. (2024) [23]	European Asian North American (White, African American, Hispanic, Asian)	↓ Vitamin D	Chronic hyperglycemia promotes the development of microvascular complications	Anjana et al. (2020) [22]
	MARD	Christensen et al. (2022) [17]	European	↑ Age ↑ BMI ↑ BP Smoking CVD history	Increased OS, changes in autophagy, retinal pigment epithelial cell damage and impaired immune responses explain the link of older age to increased risk of DR Older age is related to other risk factors for retinopathy such as hypertension	Tang et al. (2024) [24]
	MOD	Scott et al. (2024) [23]	North American (White, African American, Hispanic, Asian)	↑ BMI Ethnicity	Abdominal adiposity (assessed with WC and WHR) was associated with DR Chronic subclinical inflammation in the context of obesity may cause retinal capillary occlusion, leading to retinal ischemia and DR IR in obese individuals is related to retinal microvascular ischemia, hypoxia and increased OS, which impair blood vessel integrity, promoting DR development	Fu et al. (2023) [25]
DFS	SIDD	Xiong et al. (2021) [14] Zaharia et al. (2019) [6]	Asian European	↑ HbA1c/poor glycemic control	Hyperglycemia activates ΝF-κB, hindering leukocyte activation and migration	Moura et al. (2017) [26]
DKA	SIDD, SAID	Ahlqvist et al. (2018) [3]	European	SGLT2i treatment Early DM onset Positive family history Ethnic minority	β-cell dysfunction and insulinopenia Counter-regulatory ↑glucagon, ↑catecholamines, ↑cortisol, ↑ lipolysis and ketogenesis	Holt et al. (2021) [27]
					Impaired awareness of hypoglycemia due to AN SGLT2i are linked to euglycemic DKA	Rugg-Gunn et al. (2022) [28]

Abbreviations: *Adipo-IR* adipose tissue insulin resistance index, *AN* autonomic neuropathy, *BMI* Body Mass Index, *BP* blood pressure, *CAN* Cardiac autonomic neuropathy, *CKD* chronic kidney disease, *CVD* cardiovascular disease, *DKA* diabetic ketoacidosis, *DFS* diabetic foot syndrome, *DKD* diabetic kidney disease, *DM* diabetes mellitus, *DR* diabetic retinopathy, *DSPN* diabetic sensorimotor polyneuropathy, *ESRD* end-stage renal disease; PNPLA3 rs738409(G), G allele carrier status of the single-nucleotide polymorphism (SNP) rs738409 in the patatin-like phospholipase domain containing 3 (PNPLA3) gene; *HFpEF* heart failure with preserved ejection fraction, *HFrEF* heart failure with reduced ejection fraction, *hsCRP* high-sensitivity C-reactive protein, *ID* insulin deficiency, *IGF-1* insulin-like growth factor 1, *IGF-1R* insulin-like growth factor 1 receptor, *IHL* intrahepatic lipid, *IL-6* interleukin-6, *IR* insulin resistance, *MAD* mild age-related diabetes, *MASLD* metabolic dysfunction-associated steatotic liver disease, *MetS* metabolic syndrome, *MORD* mild obesity-related diabetes, *NF-κB* nuclear factor kappa B, *NOX5* NADPH oxidase 5, *OS* oxidative stress, PNPLA3 rs738409(G), *ROS* reactive oxygen species, *SAID* severe autoimmune diabetes, *SGLT2i* sodium-glucose cotransporter 2 inhibitor, *SIDD* severe insulin-deficient diabetes, *SIRD* severe insulin-resistant diabetes, *SNS* sympathetic nervous system, *WC* waist circumference, *WHR* waist-to-hip-ratio

As per definition, the SIRD group exhibited the highest index-based insulin resistance based on the HOMA-IR index [3], but also the lowest hyperinsulinemic-euglycemic clamp-derived insulin sensitivity [6]. Both SIRD and MOD clusters also exhibited the highest values for adipose tissue insulin resistance index (Adipo-IR) [6] and low-grade inflammation, as mirrored by high levels of high-sensitive C-reactive protein at baseline and 5 years since time of diagnosis [6].

Despite novel diabetes clusters addressing some gaps in the current classification of diabetes [1] and having been validated in large cohorts of various ethnicities [5, 9], strict classifications fail to depict the spectrum of pathophysiological alterations and their interactions, as well as the heterogeneity of diabetes [4]. Therefore, Schön et al. implemented a tree-like presentation of individuals from the GDS and the Scottish Ludwigshafen Risk and Cardiovascular Health (LURIC) cohort, using gold-standard methods such as hyperinsulinemic-euglycemic clamps to determine whole-body insulin sensitivity, and intravenous glucose tolerance tests to assess insulin secretion [4]. Hepatic lipid content was measured via ^1^H-magnetic resonance spectroscopy in the GDS cohort [4]. The study yielded similar findings to those of Zaharia et al. regarding the association of subtypes—or rather their corresponding areas in the tree structure—and diabetes complications. Participants with low insulin secretion, corresponding to the SIDD cluster, exhibited increased risk for both cardiac autonomic and diabetic sensorimotor neuropathy and a higher likelihood of needing insulin therapy, confirming previous findings (Table 1). Individuals placed in parts of the tree characterized by insulin resistance, corresponding to the SIRD subtype, exhibited the highest hepatic lipid content, markers of inflammation, increased risk for cardiovascular outcomes, depression, DFS, as well as the highest all-cause mortality. Interestingly, heart failure with preserved ejection fraction and heart failure with reduced ejection fraction exhibited distinct patterns of association with diabetes features, with the first being linked to hypertension and the latter being associated with the proportion of participants exhibiting insulin resistance, most closely resembling the SIRD subtype (Table 1). Similarly to cluster transitions over a 5-year period, ~ 20% of participants in this study altered their position across the tree-like structure, potentially related to metabolic alterations, treatment, or a combination thereof [4].

Overall, subtype-based risk stratification enables tailored complication screening and therapy [5]. SIDD patients should undergo early screening for microvascular complications and may benefit from prompt insulin therapy to preserve β-cell function [6]. SIRD patients require early renal monitoring and use of renoprotective agents regardless of baseline eGFR[6]. Their proinflammatory profile also suggests potential benefit from anti-inflammatory interventions. Tools like the Schön et al. tree model offer practical means to guide personalized management from diagnosis onward [4].

### Diabetes subtypes and cardiovascular complications

Diabetes, particularly T2D, represents a major atherogenic and cardiovascular risk factor, leading to a twofold higher risk for cardiovascular events in those with the disease than those without [29]. Within populations with T2D, however, an increased risk heterogeneity is observed [29] due to concomitant risk factors and possibly also due to distinct underlying pathophysiological mechanisms linking subtypes to cardiovascular diseases (CVDs).

Dyslipidemia is a well-established risk factor for atherosclerosis[30]. Individuals with T2D, however, exhibit a distinct dyslipidemia characterized by high triglycerides, low HDL. and small, dense LDL rather than elevated LDL cholesterol [31]. People with T2D or T1D and poor glycemic control frequently exhibit increased levels of triglyceride-rich lipoproteins, such as very low-density lipoproteins and chylomicrons, as well as their atherogenic intermediary metabolic products [32]. In a Chinese cohort, the SIDD group exhibited the highest prevalence of dyslipidemia, indicative of poor metabolic control [33]. Dyslipidemia in this group may have resulted from elevated non-esterified fatty acids causing β-cell dysfunction rather than impaired insulin sensitivity [33]. Therefore, dyslipidemia should be an early therapeutic target in this group. However, the lowest high-density lipoprotein (HDL)-cholesterol and the highest triglycerides were observed in the SIRD cluster, particularly in obese individuals with insulin resistance [33] (Table 1). Both SIRD and MOD clusters exhibited the highest fasting triglyceride levels, along with elevated high-sensitivity C-reactive protein levels, highlighting the crucial role of lipid availability and chronic subclinical inflammation in the pathogenesis of these subtypes and the development of insulin resistance [6]. Mendelian randomization studies and meta-analyses thereof have confirmed the causal role of elevated triglycerides in CVD risk [30]. Furthermore, elevated triglyceride levels are linked to residual CVD link in people treated with statins, even more so in those with diabetes [30].

The risk for CVD, major adverse cardiovascular events, and related deaths varies significantly across clusters. High CVD risk was attributed to high HbA1c and low BMI in the DEVOTE, LEADER, and SUSTAIN-6 cardiovascular outcome trials [15] most closely resembling the SAID cluster. In contrast, in the GDS, the SAID cluster exhibited the lowest cardiovascular risk scores and the SIRD group the highest ((Framingham Risk Scores for Coronary Heart Disease (FRS-CHD) and Atherosclerotic CardioVascular Disease (ASCVD) risk score) [34]. A possible explanation for these discrepancies may lie in differences in definitions and phenotyping, for instance, inclusion of individuals with differing degrees of insulin deficiency, metabolic burden, and coexisting risk factors. The triglyceride:HDL ratio, a well-established surrogate marker for insulin resistance CVD risk, was also the highest in the SIRD group [34]. As previously mentioned, people in the SIRD cluster also have the highest risk for accelerated macroalbuminuria and DKD [6], suggesting a significant cardiorenal risk in this group [35] (Table 1). Interestingly, one study found higher cardiorenal abnormalities in the SIDD and MOD group in addition to the SIRD group, while the risk burden in the MARD group was comparable to that of people with prediabetes [35]. This suggests that in the MARD group, therapeutic efforts may focus primarily on maintaining metabolic stability and preventing progression rather than intensively targeting cardiorenal complications [35]. Early prevention strategies should be tailored based on novel subtypes. In the MOD cluster, phenotypic characteristics manifest well before the clinical diagnosis of diabetes, underscoring the importance of early obesity-focused interventions for this population [35].

Therapeutic strategies addressing diabetes-induced lipid changes aim to reduce foam cell formation and vascular disease risk, particularly in insulin-resistant subtypes like SIRD [32]. Targeting endothelial insulin receptors may further alleviate endothelial dysfunction and cardiovascular complications [36]. Advanced cardiovascular risk assessment using monocyte profiling and mitochondrial function could enhance prediction in T2D [29], especially in inflammation-prone SIRD [6]. Treatments such as glucagon-like peptide-1 receptor agonists (GLP-1RAs) reduce inflammation and weight, partly via leptin [37]. Agents increasing adiponectin, including sodium-glucose cotransporter 2 inhibitors (SGLT2i) [38] and PPARγ-agonists [38], may modulate macrophage polarization and mitigate atherosclerosis [39].

### Diabetes subtypes and DFS

DFS is the leading cause of hospitalization in people with T2D [40]. The pathophysiology of DFS is complex, with key components being micro- and macrovascular damage. Diabetic sensimotor neuropathy (DSPN) impairs protective sensation, motor function (muscle wasting), and autonomic sweat production [40]. Chronic hyperglycemia impairs wound healing, prolongs inflammation, and delays tissue repair [41]. Recent evidence suggests DFS and DSPN may occur early, driven by genetic, metabolic, immune, and lifestyle factors [42].

Diabetes subtypes present distinct DFS risk profiles, shaping its pathophysiology across the diabetes spectrum (Fig. 1). SIDD carries the highest risk for DSPN [6] and microvascular complications, increasing the likelihood of developing DFS. Autonomic neuropathy in SIDD further predisposes to ulcers by reducing sweating and skin integrity [41]. Autoimmune mechanisms may contribute to DSPN in the SAID group [42], thereby potentially placing individuals in the SAID group at risk.

Microalbuminuria, hypertension, and dyslipidemia—common in SIRD—contribute to DFS and DKD risk [6]. These factors have been linked to inflammation and insulin resistance, with correlations to IL-6 and adipokines [43], with subclinical inflammation being common in SIRD and MOD [6].

The tree-like analysis in the GDS and LURIC cohorts showed that SIRD had the highest DFS and cardiovascular risk [4]. On the other hand, low BMI, which is characteristic of the SAID cluster [5], is linked to an increased risk of amputation and mortality due to diabetic foot ulcers (DFUs) [41], pointing to the association between sarcopenia and DFU [44].

Younger individuals with poor glycemic control, which is frequent in SAID, also require intensified treatment [5, 41]. However, aging [41] and frailty [44] also increase DFS risk, which is relevant to the MARD group. Aging has been linked to reduced insulin secretion and increased insulin resistance, impairing muscle structure and function [45]. Frailty and sarcopenia raise proinflammatory cytokines, such as tumor necrosis factor (TNF) and interleukin 6 (IL-6), contributing to the development of DFS [45]. Chronic hyperglycemia in SIDD may promote frailty and sarcopenia via inflammation and oxidative stress [45]. DSPN and muscle decline are bidirectionally linked: hyperglycemia weakens muscles early, while DSPN worsens it over time [45].

To conclude, distinct mechanisms underlie the increased DFS risk across diabetes clusters. SIDD is linked to neuropathy, warranting close ulcer prevention and foot screening. SIRD and MOD show DFS vulnerability through inflammation, DKD, and metabolic syndrome, requiring early vascular and inflammatory monitoring. SAID and MARD, due to sarcopenia, or frailty, need tailored management to prevent ulcers and amputation.

## MASLD and its link with diabetes mellitus and the SIRD subtype

MASLD includes hepatic fat accumulation (metabolic dysfunction-associated steatotic liver, MASL) with or without inflammation (metabolic dysfunction-associated steatohepatitis, MASH) and fibrosis, linked to at least one cardiometabolic risk factor [38]. MASLD has replaced the term nonalcoholic fatty liver disease (NAFLD) to better reflect its metabolic roots [46].

MASLD affects approximately 32% of the global adult population and is more common in people with diabetes, in 65% of those with T2D [46], and in almost 80% of individuals with both obesity and T2D [47]. In T1D, MASLD prevalence is 22% and is found particularly in people with overweight or obesity [48]. Insulin resistance is a central feature of both diabetes and MASLD [38]. As SIRD individuals exhibit a higher prevalence and risk for MASLD [5, 6], we will herein focus on the shared pathophysiological mechanisms, such as adipose tissue dysfunction and impaired mitochondrial dynamics [38].

Overnutrition leads to adipose tissue hypertrophy, local hypoxia, and inflammation [49], resulting in increased mobilization of non-esterified fatty acids (NEFAs) and glycerol [49], leading to reallocation of NEFAs to other tissues, including skeletal muscle and the liver, thus exacerbating whole-body and hepatic insulin resistance and driving MASLD progression [50]. Interestingly, obese individuals with MASLD exhibit lower mitochondrial respiration selectively in visceral, but not subcutaneous, adipose tissue, which is linked to reduced adipose tissue insulin sensitivity and adipose tissue dysfunction [51], while similar impairments may occur in skeletal muscle [52].

Muscle insulin resistance shifts substrate use toward lipid metabolism, fueling hepatic de novo lipogenesis (DNL) and gluconeogenesis [49]. These changes contribute to hepatic insulin resistance, increased endogenous glucose production [49], and fasting hyperglycemia [53], further worsening MASLD [38]. Notably, in early obesity and MASLD, an attempt to counteract the increased NEFA flux to the liver through an increase in hepatic mitochondrial respiration occurs; however, this plasticity is lost upon progression to MASH [54], specifically in the presence of prediabetes [55] and T2D [56], leading to decreased β-oxidation.

Obesity intensifies MASLD in individuals with diabetes caused by chronic inflammation and fibrosis development [57]. In people with obesity and T2D, liver disease progression is significantly accelerated [58]. In contrast, T2D does not appear to exacerbate MASLD progression in lean patients, suggesting a BMI-dependent effect [59]. Weight loss, especially in younger persons, reduces the risk of MASLD, at-risk-MASH, and liver stiffness [60], although the pathophysiological mechanisms remain incompletely understood.

Fibrosis is the strongest predictor of adverse liver outcomes [61]. T2D and the SIRD cluster are associated with a higher risk of advanced fibrosis, which is linked to mitochondrial dysfunction, oxidative stress, and altered adipokine secretion [6, 38, 58].

Genetics also play a role. The PNPLA3 rs738409 G allele increases MASLD risk and fibrosis progression [62] and is more prevalent in SIRD [21] where it is associated with adipose tissue insulin resistance [21]. This variant also influences the metabolic response to dietary interventions, underscoring the importance of personalized treatment approaches for individuals with MASLD and T2D [63].

MASLD involves inter-organ crosstalk among adipose tissue, liver, and skeletal muscle [8]. Adipose tissue dysfunction initiates a cascade of metabolic disturbances leading to systemic insulin resistance and MASLD progression [49]. Exosomes released from hepatocytes, adipocytes, and muscle cells contribute to hepatic inflammation, fibrosis, and insulin resistance [8]. Furthermore, exosomes secreted by lipotoxic hepatocytes are involved in the development of fibrosis, potentially through downregulating mitophagy and promoting hepatic stellate cell activation [64]. Exosomal microRNAs (miRNAs) are emerging biomarkers in MASLD and diabetes and may represent future therapeutic targets [8], although several research questions remain unanswered, including adipose tissue depot-specific exosomes effects for both diabetes and MASLD.

Circulating levels of fibroblast growth factor 21 (FGF21) and growth differentiation factor 15 (GDF15) are elevated in MASLD and T2D [8]. FGF21 improves insulin sensitivity and lipid metabolism [8], while GDF15 regulates appetite and has direct metabolic effects, making them potential therapeutic agents [65].

The gut-brain-liver axis and intestinal microbiota are also involved in MASLD pathogenesis [38]. Individuals with MASLD and T2D show gut dysbiosis [38], increased intestinal permeability [49], and endotoxin translocation, which promotes systemic and hypothalamic inflammation [8]. Dysbiosis also alters bile acid metabolism [61], disrupting glucose and lipid homeostasis through effects on farnesoid X receptor (FXR) and fibroblast growth factor 19 (FGF19) signaling pathways. These changes contribute to liver injury and disease progression[38].

Different diabetes subtypes show varying levels of inflammation and risk for MASLD (Fig. 1), reflecting the heterogeneity of underlying disease mechanisms. The SIRD subtype exhibits the highest levels of systemic inflammation, as indicated by biomarkers such as high-sensitivity C-reactive protein (hsCRP)[6], caspase 8 (a mediator of apoptosis, including β-cell death), and markers of immune cell activation, including B cells, T cells, and natural killer cells [8]. These findings highlight how chronic inflammation drives insulin resistance and liver disease in SIRD. As such, anti-inflammatory strategies or treatments aimed at improving insulin sensitivity may be particularly effective for patients within the SIRD cluster.

Conversely, the SIDD group exhibits decreased levels of caspase 8 and interleukin 6 (IL-6) [66], pointing to a less proinflammatory state in this cluster, this further underlining the view that the heterogeneous spectrum of diabetes mellitus cannot be attributed solely to adipose tissue dysfunction and its associated cross-talk [8].

Proteomic and metabolomic studies show that subtype differences are clinically relevant and rooted in distinct biological pathways [8]. Recent research has demonstrated that even in prediabetes, individuals exhibit divergent metabolic profiles depending on future diabetes subtype, including differences in hepatic steatosis, inflammation, and metabolic responses [67]. This suggests that inter-organ crosstalk and metabolic dysregulation begin well before the onset of overt diabetes, thus highlighting the potential of early identification and prevention strategies in high-risk cases [8].

### Clinical impact of MASLD in diabetes and SIRD

Coexistence of MASLD with T2D and other aspects of metabolic dysfunction places individuals at a higher risk of liver disease progression to MASH, cirrhosis, and HCC, in addition to extrahepatic outcomes such as cardiovascular and kidney disease [61]. T2D and MASLD comprise established and independent risk factors for cardiovascular disease (CVD) [68], irrespective of other common cardiovascular risk factors [8, 57]. Intrahepatic lipid accumulation and development of MASLD elicit alterations in lipid and lipoprotein metabolism [38], including an increase in circulating triglycerides and low-density lipoprotein (LDL) levels [68]. These alterations accelerate the progression of cardiovascular and cardiorenal risks far in advance of overt T2D manifestation and development of hyperglycemia [68]. CVD-related mortality appears to be the leading cause of death in individuals with MASLD, although liver-related mortality increases with advanced fibrosis and cirrhosis [46]. This relationship seems to be reversed in people with non-cirrhotic liver disease in whom extrahepatic cancers and CVD are the leading causes of death [49]. The complex multidirectional interplay between various metabolic risk factors, particularly T2D and MASLD, significantly increase morbidity and mortality [61].

### MASLD as a therapeutic target in diabetes and SIRD

The bidirectional relationship between MASLD and SIRD/T2D underscores the importance of improving steatosis, fibrosis, and hepatic insulin resistance as crucial therapeutic targets in individuals with diabetes (Fig. 1).

Diabetes increases the need for MASLD screening [69], especially in the high-risk SIRD cluster [6]. Clusters can be identified using tools such as the diabetes-cluster-tool [70], https://diabetescalculator.ddz.de/diabetescluster-en/, which uses simple clinical parameters to identify high-risk SIRD individuals and intensify monitoring and treatment [69]. Regular screening for MASLD and fibrosis is recommended in SIRD/T2D to prevent progression to cirrhosis and complications [69]. Lifestyle strategies remain the cornerstone of MASLD management [69], exerting beneficial effects on steatosis, insulin resistance, and glycemia through weight loss-dependent and independent mechanisms [71]. While a 5% weight loss improves steatosis and 7–10% is needed for inflammation and ≥ 10% for fibrosis, achieving and maintaining these remains key in MASLD management [69]. Dietary approaches targeting MASLD and MASH in people with T2D and with or without overweight or obesity involve simple caloric restriction [72], manipulation of meal timing [73], and alterations of diet composition [74]. Current clinical guidelines for the management of MASLD recommend limiting the intake of ultra-processed foods and fructose-sweetened beverages, adhering to a Mediterranean-like dietary pattern, and engaging in regular physical activity [69]. In particular, the Mediterranean diet has the most pronounced effects on MASLD, significantly reducing intrahepatic lipid content and fibrosis [38] while additionally improving insulin sensitivity and secretion, independently of weight loss [72]. These features are particularly relevant in individuals within the SIRD cluster in whom systemic insulin resistance and inflammation are most pronounced [6].

Combined aerobic and resistance training improves glycemia and reduces hepatic steatosis, especially when combined with caloric restriction [57]. Reductions in hepatic lipid content through exercise training are associated with an improvement in skeletal muscle insulin sensitivity but not with changes in adipose tissue or hepatic insulin sensitivity and seem to be mainly driven by weight loss [75]. Chronic endurance and resistance exercise may prevent the development of insulin resistance, dyslipidemia, and T2D[76] potentially through inducing mitochondrial adaptations, including increases in oxidative capacity and lipid oxidation [76]. While effective lifestyle strategies have limited sustainability, they are less efficacious than emerging pharmacotherapies for obesity, T2D, and MASLD[57].

Before incretin therapies, bariatric surgery was the most effective approach for ~ 35% weight loss [77], improving hepatic outcomes, glycemia, insulin sensitivity, and adipokine profiles [57, 71, 78]. Bariatric surgery leads to significant weight loss, remission of T2D [38], and reduction of steatosis, MASH, and fibrosis[79] as well as being associated with cardiovascular and cardiorenal benefits [78]. The bariatric-metabolic surgery versus lifestyle intervention plus best medical care in what was previously termed non-alcoholic steatohepatitis (now renamed MASH) (BRAVES) randomized control trial, demonstrated the superior efficacy of Roux-en-Y gastric bypass or sleeve gastrectomy compared to lifestyle interventions with regard to histological resolution of MASH [80]. Surgery-induced weight loss improves systemic and tissue-specific insulin sensitivity in skeletal muscle [52] and adipose tissue [81] and reduces chronic low-grade inflammation [71]. Bariatric surgery appears to outperform T2D pharmacotherapies, including lifestyle advice, weight control, glucose monitoring, and FDA-approved incretin mimetics [82]. Furthermore, metabolic surgery reduces the risk for both MASLD- and T2D-related cancers [83]. Nevertheless, the invasiveness and proportionally high loss of fat-free mass [77] as well as the short- and long-term side effects of such procedures present the need for the development and use of novel therapies of less invasive nature with comparable efficacy [57].

Novel incretin-based therapies, initially developed for the treatment of diabetes mellitus due to their insulinotropic effects in the presence of hyperglycemia [84], have now emerged as potent antiobesity drugs while also showing great therapeutic potential for MASLD and MASH [57]. GLP-1RAs delay gastric emptying, decrease glucagon secretion, and act centrally to reduce appetite and food intake, subsequently leading to weight loss [57]. Semaglutide [85] and liraglutide [86] have demonstrated improvements in steatosis and MASH resolution; however, so far, they have not shown any histologically confirmed significant improvements in fibrosis [87]. The first results of the ongoing phase III ESSENCE trial, evaluating once-weekly semaglutide 2.4 mg treatment in participants with biopsy-proven MASH and fibrosis stage F2 or F3, addresses this gap by demonstrating improvements in both steatohepatitis and hepatic fibrosis in about 37% of participants receiving semaglutide [88].

Combination therapies, including dual GLP-1 and glucagon receptor agonists (GCG/GLP-1RA) such as pemvidutide [89] and cotadutide [90], which have been investigated in phase IIa and III studies, respectively, dual GLP-1/GIP-RA such as tirzepatide or triple GCG/GIP/GLP-1RAs such as retatrutide, whose efficacy was tested in phase II and IIa trials, respectively, have reported promising results. An exploratory study showed significant reductions in biomarkers of MASH and fibrosis in a population with T2D, although these results should be confirmed in further studies including participants with liver biopsy [91]. Of note, polyagonist treatment results in more reductions in HbA1c and bodyweight in T2D compared to GLP-1RAs [61], while pemvidutide treatment may additionally enhance energy expenditure [89] (Table 2). Novel incretin-based therapies in phase II trials achieved a weight loss of up to 24.2% according to a recent systematic review [93]. According to a network meta-analysis of available randomized control trials, of all available lifestyle and pharmacological treatments for MASLD, GLP-1RΑs are the most efficient in improving liver-related outcomes [94]. However, their primarily gastrointestinal-related side effects and high costs pose major limitations [57].

**Table 2:** Mechanistically characterized, peer-reviewed randomized trials in MASLD and type 2 diabetes: human studies on pharmacotherapies with non-invasive or biopsy-based diagnosis only

Study	Year	Study type	Population characteristics	Intervention (n)	Mechanistic insights	Therapeutic strategies
*Currently available pharmacotherapies*						
Liu et al. (2025) [92]	2025	Cohort study	T2D + MASLD (MRI-PDFF)	Dulaglutide 1.5 mg QW (38)	-GLP-1RA-mediated IHL reduction may promote recovery of pancreatic islet function, as mirrored in improved postprandial C-peptide secretion	GLP-1RA
Harrison et al. (2024) [87]	2024	RCT	MASH and fibrosis (liver biopsy)	Resmetirom 80 mg (322) vs. 100 mg (323) vs. PL (321)	- ↑ MASH resolution and ↓ fibrosis reduction compared to placebo - direct hepatic effects unrelated to weight loss - ↓ inflammation - ↓LDL levels (↓ cholesterol production or ↑ lipid clearance)	Thyromimetics
Kahl et al. (2020) [83]	2020	RCT	T2D ± MASLD (^1^H-MRS)	Empagliflozin 25 mg QD (42) vs. PL (42)	- ↓ IHL - primarily indirect mechanisms related to weight loss - no direct hepatic or anti-inflammatory effects - ↑ whole-body insulin sensitivity, driven mostly by skeletal muscle - ↔ hepatic insulin sensitivity - ↔ adipose tissue insulin sensitivity	SGLT2i
*Novel pharmacotherapies*						
Harrison et al. (2025) [84]	2025	RCT	T2D + MASH with fibrosis (F1-F3) (MRI-PDFF) on GLP-1RA	GLP-1RA (semaglutide 1 mg QW or dulaglutide 3 mg QW or liraglutide 1.5 mg) QW + PL (10) vs. GLP-1RA + efruxifermin 50 mg QW (21)	- ↓ food intake, ↓ bodyweight - ↓ IHL, ↓ SH (weight loss-dependent) - ↓ fibrosis - ↓ markers of fibrogenesis and hepatocellular injury independently of antisteatotic effects -↓ markers of fibrosis, liver injury - ↑ markers of insulin sensitivity	Fc-FGF21 analog (efruxifermin)
Harrison et al. (2025) [77]	2025	RCT	OWE/OBE + MASLD (MRI-PDFF) ± T2D	Pemvidutide 1.2 mg (23) or 1.8 mg (23) or 2.4 mg (24) vs. PL (24)	- ↓ steatosis - ↓ non-invasive markers of fibrosis in a dose-dependent manner - Hepatic effects are indirect and related to weight loss - ↑ weight loss compared to GLP-1Ra - ↑ energy expenditure	Dual GLP-1R/GCGR agonism
Loomba et al. (2024) [89]	2024	Multicenter RCT	MASH with advanced fibrosis (≥ F2)	Denifanstat 50 mg (112) vs. PL (56)	- ↓ DNL - ↓ steatosis - ↓ inflammation - ↓ fibrosis - ↑ MASH resolution compared to placebo - additional cardiovascular benefits: ↓ LDL cholesterol, ↓ PUFA, but ↑ TG	Oral fatty acid synthase inhibitor (FASN)
Parker et al. (2023) [78]	2023	RCT	OWE/OBE + T2D ± MASLD (MRI-PDFF)	Cotadutide titrated 50–300 μg (12) vs. liraglutide titrated 0.6–1.8 (10) vs. PL (9)	- ↓ IHL, ↓ hepatic glycogen compared to liraglutide or placebo - ↑ glycemic control, ↓ body weight, ↓ dyslipidemia and ↓ inflammation - ↑ hepatic FAO and ↓ glycogenolysis through GCG agonism compared to GLP-1R agonism alone -Effects on hepatic glycogen unrelated to weight loss - GLP-1R expression is absent in the liver and hepatic are rather indirect and mediated through weight loss	Dual GLP-1R/GCGR agonism

Abbreviations: ^1^H-MRS, proton magnetic resonance spectroscopy, *Adipo-IR* adipose insulin resistance index, *BMI* body mass index, *CON* control, *FAO* fatty acid oxidation, *Fc-FGF21* fusion protein of the Fc fragment of immunoglobulin G (IgG) and fibroblast growth factor 21, *FGF21* fibroblast growth factor 21, *GCGR* glucagon receptor, *GLP-1* glucagon-like peptide-1, *GLP-1RA* glucagon-like peptide-1 receptor agonist, *HOMA-IR* homeostasis model assessment of insulin resistance, *IHL* intrahepatic lipid, *IR* insulin resistance, *LDL* low-density lipoprotein, *MASH* metabolic dysfunction-associated steatohepatitis, *MASLD* metabolic dysfunction-associated steatotic liver disease, *MRI-PDFF* magnetic resonance imaging-proton density fat fraction, *MRI* magnetic resonance imaging, *OBE* obesity, *OWE* overweight, *OXPHOS* oxidative phosphorylation, *PL* placebo, *QD* once-daily, *QW* once-weekly, *RCT-X* randomized crossover trial, *RCT* randomized controlled trial, *SAT* subcutaneous adipose tissue, *SGLT2i* sodium-glucose co-transporter 2 inhibitors, *T1D* type-1 diabetes, *T2D* type-2 diabetes, *TG* triglyceride, *VAT* visceral adipose tissue, *WC* waist circumference

Sodium glucose linked transporter 2 inhibitors (SGLT2i) induce glucosuria through blockage of the proximal renal tubular glucose reabsorption, resulting in a negative energy balance and thereby reductions in body weight and hyperglycemia [95]. SGLT2i target MASLD through weight loss, but also in weight loss-independent mechanisms involving improvements in inflammation and insulin resistance. Empagliflozin has been shown in a phase IV trial to reduce intrahepatic lipid content [96], associated with improvements in adiponectin levels [96], however, with limited effects on fibrosis risk [38]. The therapeutic potential of SGLT2i in MASH, specifically, is rather limited [96], although treatment with SGLT2i in T2D have established cardiovascular and cardiorenal outcomes and should therefore be preferred for populations with these comorbidities [38]. Both SGLT2i and GCG/GLP-1RA may be especially relevant for SIRD individuals, who exhibit high hepatic lipid content and are at greater risk for MASLD-related fibrosis and cardiorenal complications [6].

FGF21 analogs target adipose tissue inflammation and insulin resistance, which are key factors in both T2D and MASLD [71, 97]. FGF21 is involved in lipid and glucose metabolism regulation [71]. Treatment with the FGF21 analog pegozafermin is associated with improvements in steatosis, fibrosis, and glycemia in participants with MASH, as shown in a recent phase IIb trial [71]. A recent phase IIb study in patients with T2D and MASH showed that treatment with the Fc-FGF21 analog efruxifermin in addition to GLP-1RA treatment led to further improvements in steatosis and non-invasive markers of fibrosis and glucose metabolism compared to GLP-1Ra monotherapy, suggesting additive effects [97] (Table 2). FGF21 analogs like efruxifermin improve liver outcomes largely independently of weight loss, suggesting direct hepatic effects and synergy with GLP-1RAs [97] (Table 2). However, in a recent phase IIb study involving participants with biopsy-confirmed compensated cirrhosis (F4), efruxifermin treatment did not result in significant improvements in fibrosis [98].

Resmetirom, the selective hepatic thyroid hormone receptor (THR)-β agonist, the first FDA-approved drug for the treatment of non-cirrhotic MASH and liver fibrosis (F ≥ 2), improves lipid utilization in the liver through promoting β-oxidation [69]. Specifically, thyroid hormones regulate triglyceride and cholesterol metabolism in the liver and promote autophagy-mediated lipolysis of triglycerides stored in lipid droplets, referred to as “lipophagy” [99]. Thyroid hormones regulate blood glucose levels through their hepatic actions, specifically through increasing glycogenolysis and gluconeogenesis, resulting in a net increased hepatic glucose output, which is compensated by increased skeletal muscle glucose uptake [99]. However, both hyper- and hypothyroidism have been linked to insulin resistance and MASLD [100], possibly pointing to the thyrotropic axis as a key player and therapeutic target. Additionally, thyroid hormones promote mitochondrial biogenesis and mitophagy in order to preserve adequate hepatic oxidative capacity [99]. In a phase III trial, resmetirom demonstrated efficacy on histologically confirmed MASH and fibrosis [100]. Of note, improvements in fibrosis were not related to the presence of diabetes, fibrosis stage, or concomitant weight loss during the treatment period [100]. Resmetirom improves MASH and fibrosis while lowering LDL, reflecting effects on hepatic cholesterol and lipid clearance [100] (Table 2). Future studies should explore the efficacy and safety of combinations of resmetirom and incretin mimetics, particularly in people with obesity and diabetes.

Peroxisome proliferator-activated receptors (PPARs), which are nuclear receptors involved in the regulation of energy homeostasis with distinct and overlapping actions, have surfaced as promising targets for the treatment of MASLD and diabetes mellitus [49, 101]. PPARα is primarily involved in the regulation of lipid metabolism, whereas PPARβ/δ activation triggers the anti-inflammatory polarization of hepatic macrophages [101]. PPARγ agonists, such as thiazolidinediones, are well-established insulin sensitizers and have been implemented in the treatment for T2D for an extended period of time. The PPARγ-agonists pioglitazone and rosiglitazone are effective in improving steatosis and glycemia; however, available studies have reported varied effects on fibrosis and MASH resolution, with some showing improvement while others demonstrated no significant effect [71]. Elafibranor, a dual PPAR-α and -δ agonist, and the pan-PPAR agonist lanifibranor led to resolution of MASH without fibrosis deterioration in phase IIb trials [71].

Denifanstat, a fatty acid synthase inhibitor, shows promise in treating MASH by reducing DNL and liver steatosis, thereby mitigating inflammation and fibrosis [102]. A phase IIb trial revealed MASH resolution and fibrosis improvement [102] (Table 2). By lowering DNL, it reduces mitochondrial stress, restoring function and reducing ROS and inflammation [38, 54]. A concern with DNL suppression in MASLD is the shift of lipids to harmful pathways, such as increased blood lipids [49]. Denifanstat raised triglycerides but also lowered LDL and PUFA levels, offering some cardiovascular benefits [102].

In summary, MASLD is a key target in SIRD in which insulin resistance, adipose dysfunction, and inflammation drive hepatic and systemic metabolic deterioration [6]. Given their elevated risk for fibrosis and cardiometabolic complications, individuals with SIRD may benefit most from intensified MASLD screening and early initiation of combination therapies. Emerging agents such as GLP-1/GIP polyagonists [87], FGF21 analogs [38], and SGLT2 inhibitors [96], alongside lifestyle intervention [57, 71], may offer synergistic benefits in this high-risk group. Future trials should consider diabetes subtype stratification to optimize treatment outcomes.

## Limitations of phenotype-based clustering approaches

While the recent classification of the newly proposed diabetes subtypes, such as SIDD, SIRD, MOD, MARD, and SAID, offers insights into heterogeneity, clinical implementation remains limited by methodological challenges (Fig. 1).

One key issue is the possible instability over time [5], as shown by about 23% of persons changing their subtype within 5 years [6]. Some input variables also lack standardization and/or are incomplete, e.g., for diagnosis of autoimmune diabetes. Indeed, the original classification using GADA antibodies to identify SAID [3] leads to lower prevalence of SAID when compared to using additional autoantibodies, this misclassification occurring in about 7% of cases [6].

Subgroups are not mutually exclusive, with overlapping features that challenge clinical utility. Alternative clustering frameworks, such as continuum-based models, may better reflect diabetes pathophysiology [4].

Evidence from randomized trials is also lacking. Subtype-specific treatment responses have been observed retrospectively, but prospective validation is missing [9, 19, 103–105]. A clear link to prediabetes subtypes is currently also missing [9].

Ethnic and geographic generalizability is additionally limited. Most models were developed in European cohorts and show inconsistent replication elsewhere [9].

Integrating genetic and phenotypic data presents further complexity [9]. Combining genotype and phenotypic data may enhance subtyping, but integration remains unresolved due to phenotypic variability [10].

Moreover, there is a lack of direct comparisons with alternative predictive models [10, 11]. Continuous-variable models may outperform current clusters in outcome prediction [3, 4, 6, 9]. Finally, implementation in practical clinical care poses challenges [9]. Even if subtype-based approaches evolve, practical use will require clear, actionable guidance tailored to diagnostics and treatment [9].

## Conclusions

Diabetes mellitus is a heterogeneous multisystem disorder driven by overlapping mechanisms. Subtype classifications move beyond the traditional dichotomy into type 1 and type 2 diabetes [3, 4, 6].

Herein we have highlighted the SIRD subtype due to its strong association with insulin resistance and elevated risk for complications including MASLD, CVD, and DFS [4, 6]. Though less prevalent, SIRD reflects the rising global burden of obesity and diabetes and aligns with the therapeutic targets of emerging multi-agonist and incretin-based treatments, although weight loss remains a critical therapeutic goal in MASLD, improving both liver histological features and insulin resistance [57], and can be achieved through lifestyle interventions encompassing both exercise and dietary modification [69], in addition to novel pharmacological options [57, 71, 94].

We also examined diabetes-related complications across subtypes, with a focus on conditions where clinical progression is influenced by metabolic dysfunction. DFS was selected as an illustrative example, integrating vascular and metabolic contributors across clusters [40, 41, 43, 44]. Further complications such as retinopathy were mentioned but not discussed in depth as they have already been well-characterized [6, 23] and thoroughly discussed in recent reviews [13].

SAID and SIDD also progress rapidly due to β-cell failure, leading to complications including neuropathy, cardiovascular disease, and retinopathy [3, 4, 6, 15, 22].

Understanding subtype-complication links will improve risk stratification and care [5]. The key message of this review is that diabetes subtypes differ not only in metabolic profiles but also in their propensity for specific complications [4–6]. Recognizing these differences may have direct clinical implications for prevention, screening for early complication, guiding therapy selection, and monitoring disease progression.

In summary, current phenotype-based clustering shows great promise, but also raises some open questions. Subtyping offers a conceptual framework to inform clinical decisions, especially when paired with clinical judgment and evolving biomarker tools to enable future targeted diabetes care.

## Data Availability

This is a review article and does not contain any original data. All data cited are available from the referenced publications.

## Abbreviations

Adipo-IR: Adipose tissue insulin resistance index
AGE: Advanced glycation end-product
BMI: Body mass index
CAN: Cardiac autonomic neuropathy
CVD: Cardiovascular disease
DFS: Diabetic foot syndrome
DFU: Diabetic foot ulcer
DKA: Diabetic ketoacidosis
DKD: Diabetic kidney disease
DM: Diabetes mellitus
DR: Diabetic retinopathy
DSPN: Distal symmetric polyneuropathy
Egfr: Estimated glomerular filtration rate
ESRD: End-stage renal disease
FGF19: Fibroblast growth factor 19
FGF21: Fibroblast growth factor 21
FXR: Farnesoid X receptor
GDF15: Growth differentiation factor 15
GLP-1RA: Glucagon-like peptide-1 receptor agonist
HbA1c: Hemoglobin A1c
HDL: High-density lipoprotein
HOMA2-B: Homeostasis Model Assessment 2 – Beta Cell Function
HOMA2-IR: Homeostasis Model Assessment 2 – Insulin Resistance
hsCRP: Hgh-sensitivity C-reactive protein
IL-6: Interleukin 6
IR: Insulin resistance
LDL: Low-density lipoprotein
MARD: Mild age-related diabetes
MASH: Metabolic dysfunction-associated steatohepatitis
MASL: Metabolic dysfunction-associated steatotic liver
MASLD: Metabolic dysfunction-associated steatotic liver disease
MOD: Mild obesity-related diabetes
NEFA: Non-esterified fatty acid
NF-κB: Nuclear factor kappa B
PNPLA3: Patatin-like phospholipase domain-containing protein 3
PPARγ: Peroxisome proliferator-activated receptor gamma
ROS: Reactive oxygen species
SAID: Severe autoimmune diabetes
SGLT2i: Sodium-glucose cotransporter 2 inhibitor
SIDD: Severe insulin-deficient diabetes
SIRD: Severe insulin-resistant diabetes
T1D: Type 1 diabetes
T2D: Type 2 diabetes
TNF: Tumor necrosis factor
